# Effects of Steroid Hormone Levels on the Ultrasound Appearance of the Preovulatory Endometrium in Controlled Ovarian Hyperstimulation Cycles 

**Published:** 2012-03-20

**Authors:** Aytul Corbacioglu, Bulent Baysal

**Affiliations:** Department of Obstetrics and Gynecology, Istanbul University Istanbul Faculty of Medicine, Istanbul, Turkey

**Keywords:** Endometrium, *In vitro* fertilization, Ultrasound, Estradiol, Progesterone

## Abstract

**Background:**

This study investigated the effects of serum estradiol (E2) and progesterone levels on preovulatory endometrial thickness and echogenicity in controlled ovarian hyperstimulation (COH) cycles for *in vitro* fertilization (IVF).

**Materials and Methods:**

In this retrospective study, we evaluated the data of 241 *in vitro* fertilization- embryo transfer cycles. Cycles were classified into three groups according to endometrial thickness measured on the day of human chorionic gonadotropin (hCG) administration as: i. ≤8 mm, ii. 8-14 mm, and iii. ≥14 mm. Echogenic patterns were described as trilaminar, isoechogenic, and hyperechogenic. Serum E2 and progesterone levels were evaluated on the day of hCG administration. Data were analyzed using X2test, Student’s t test and analysis of variance (ANOVA).

**Results:**

Serum E2 levels increased in parallel with endometrial thickness, although differences among the three groups were insignificant. There was no correlation between serum progesterone levels and endometrial thickness. There was no significant difference in the steroid hormone concentrations between the echogenic patterns.

**Conclusion:**

Serum steroid hormone levels on the day of hCG administration do not affect the ultrasound appearance of the endometrium in COH cycles.

## Introduction

Endometrial receptivity is one of the most important factors in the implantation process. The role of an ultrasonographic evaluation of the endometrium in predicting the implantation rate has long been debated. Although many studies consider a trilaminar echogenic pattern and endometrium thickness within a definite range as favorable prognostic factors (1), recent investigations have shown that the ultrasonographic appearance of the endometrium does not predict pregnancy outcome (2).

Estrogen is essential for implantation because of its effects on both endometrial proliferation and the augmentation of uterine perfusion. Although its concentration has been predicted to be associated with endometrial thickness, the results of investigations are conflicting (3, 4). Another controversial issue is the association between steroid hormones and endometrial echogenicity. In natural cycles, while a hypoechogenic pattern is observed during the proliferative phase, the hyperechogenic endometrium is a feature of the secretory phase. Investigations have shown a relationship between the serum progesterone level and secretory changes in the endometrium in controlled ovarian hyperstimulation (COH) cycles (5). However, some studies have demonstrated no correlation between high progesterone levels and a hyperechogenic endometrial pattern on the day of human chorionic gonadotropin (hCG) administration (6).

Thus, we aimed to investigate the effects of serum estradiol (E2) and progesterone levels on preovulatory endometrial thickness and echogenicity in COH cycles for *in vitro* fertilization.

## Materials and Methods

In this retrospective study approved by the Institutional Review Board (IRB) of Istanbul Faculty of Medicine, we evaluated the data of 241 *in vitro* fertilization-embryo transfer cycles performed at the Reproductive Endocrinology Unit of the Istanbul Faculty of Medicine between February 2005 and Febuary 2007. We performed either the long gonadotropin-releasing hormone (GnRH) agonist or GnRH antagonist protocols for stimulation according to the patient’s age, ovarian reserve, and previous response to ovarian stimulation, preferring the antagonist protocol for poor responders. We excluded the cycles in which an embryo transfer was not performed or E2 was administered with the aim of improving endometrial thickness. Also, we included only the first cycle if a patient underwent two or more cycles during this period. In the long agonist protocol, 1 mg of leuprolide acetate (Lucrin, Japan) S.C. was started on the 21st day of the previous cycle. After the baseline ultrasonographic evaluation of the endometrium and ovaries on the third day of the cycle, either recombinant FSH (Gonal-F; follitropin alfa, or Puregon; follitropin beta) or human menopausal gonadotropin (hMG) was started for ovarian stimulation at an appropriate dosage, and the dose of the GnRH agonist was reduced by half. In the antagonist protocol, the GnRH antagonist cetrorelix (Cetrotide, Germany) at a dose of 0.25 mg/day was started when at least one follicle reached 14 mm in diameter and was continued daily until administration of the hCG ovulation-trigger dose. A total of 10000 U hCG (Profasi or Pregnyl) was administered when at least two follicles were 18 mm in diameter. Oocyte retrieval was performed approximately 36 hours after hCG administration by transvaginal ultrasound guided follicular aspiration. Grades 1 and 2 embryos were replaced 3 days after oocyte retrieval. Pregnancy was diagnosed by serum β-hCG concentrations on the 12^th^ day of embryo transfer.

Endometrial thickness and the echogenic pattern were evaluated on the day of hCG administration by transvaginal ultrasound, performed by four specialists. Endometrial thickness was defined as the greatest anteroposterior dimension in the sagittal plane. The cases were classified in three groups according to the measurement of endometrial thickness: ≤8 mm, 8-14 mm, and ≥14 mm. Echogenic patterns were described as trilaminar (hypoechogenic endometrium with central and outer echogenic lines), isoechogenic (same reflectivity compared to myometrium), and hyperechogenic (increased reflectivity compared to myometrium). Serum E2 levels in pg/ml and progesterone levels in ng/ml were evaluated on the day of hCG administration as a routine procedure in our clinic.

Data were analyzed using the χ2 test, Student’s t test, and analysis of variance (ANOVA), using statistical package for Windows, (version 9.0) (SPSS Inc., Chicago, IL). A p value of <0.05 was considered significant.

## Results

The patient characteristics are summarized in table 1. Endometrial thickness was measured as ≤8 mm (group 1) in 51 cases, 8-14 mm (group 2) in 182 cases, and ≥14 mm (group 3) in 8 cases (Table 2). Serum E2 levels increased in parallel with the endometrial thickness, although differences among the three groups were insignificant. There was no significant difference between the number of retrieved oocytes and transferred embryos.

**Table 1:** Summary of demographic data


**Total number of ART cycles**		241
Age (years)*		31.3 ± 5.4
Duration of infertility (years)*		7.3 ± 4.5
**Diagnosis (%)**		
	**Male factors**	45.6
	**Tuboperitoneal factors**	17
	**Ovarian factors**	5
	**Endometriosis**	2.5
	**Unexplained**	26.1
	**Multiple**	3.7
Number of ART attempts*		1.5 ± 1


* Values are mean ± SD.

The echogenic patterns are compared in table 3. Patients were older and the total gonadotropin dose was higher in the hyperechogenic group. However, we could not find a significant difference among the steroid hormone concentrations on the day of hCG administration.

Among the 241 COH cycles, 64 cycles resulted in pregnancies, with a total pregnancy rate of 26.6%.

**Table 2:** Comparisons of variables between the groups according to endometrial thickness


Variable	Group 1 (n = 51)	Group 2 (n = 182)	Group 3 (n = 8)	P-value

Age (years)*	31.33 ± 5.09	31.38 ± 5.55	31.50 ± 5.86	0.99
Total dose of gonadotropin (IU)*	3025.39 ± 1379.53	3308.58 ± 1329.41	3309.37 ± 2031.31	0.42
hCG day E2 (pg/mL)*	3079.75 ± 2010.33	3090.45 ± 1815.77	3232.33 ± 2617.99	0.98
hCG day progesterone (ng/mL)*	1.40 ± 1.06	1.52 ± 1.46	1.06 ± 0.33	0.70
Retrieved oocytes (n)*	13.43 ± 7.81	12.71 ± 7.03	12 ± 8.7	0.77
Transferred embryos (n)*	2.71 ± 0.64	2.80 ± 0.50	2.63 ± 0.52	0.37
**Pregnancy rate (%)**	29.4	26.4	12.5	0.59


* Values are mean ± SD.

**Table 3:** Comparisons of variables between the groups according to endometrial echogenicity


Variable	Trilaminar (n = 184)	Isoechogenic(n = 24)	Hyperechogenic (n = 33)	P-value

Age (years)*	30.82 ± 5.33	33.04 ± 5.46	33.27 ± 5.57	0.01
Total dose of gonadotropin (IU)*	3133.32 ± 1334.31	3467.79 ± 1202.42	3731.06 ± 1549.09	0.04
hCG day E2 (pg/mL)*	3112.40 ± 1678.30	2835.17 ± 2307.44	3190 ± 2552.42	0.79
hCG day progesterone (ng/mL)*	1.50 ± 1.38	1.81 ± 1.84	1.12 ± 0.59	0.25
Retrieved oocytes (n)*	13.54 ± 7.37	11.79 ± 5.62	9.70 ± 6.72	0.01
Transferred embryos (n)*	2.81 ± 0.49	2.83 ± 0.48	2.55 ± 0.71	0.02
**Pregnancy rate (%)**	26.6	20.8	30.3	0.72


* Values are mean ± SD.

The serum E2 levels were 3166.89 ± 1713.02 pg/ml in the conception cycles and 3066.70 ± 1930.27 pg/ml in the non-conception cycles. Serum progesterone levels were 1.48 ± 1.32 ng/ml in the conception and 1.48 ± 1.39 ng/ml in the non-conception cycles. There were no significant differences between conception and non-conception cycles in the means of steroid hormone levels.

## Discussion

The endometrium is a highly dynamic tissue changing cyclically in response to steroid hormones in order to create a window of receptivity for implantation (7). The ultrasound appearance of the endometrium reflects these cyclic changes. In the postmenstrual period, the endometrium is observed as a thin, regular, echogenic line. As a result of the straight, orderly arrangement of glandular elements in the proliferative phase, the endometrium is relatively hypoechoic with a central echogenic line reflecting the collapsed endometrial lumen. It reaches 8–14 mm in the peri-ovulatory phase. In the secretory phase, the endometrium achieves its maximum thickness and echogenicity because of distended, tortuous glands that contain secretions. In spontaneous cycles, endometrial appearance is correlated with increasing E2 and progesterone levels, while the results of studies of COH cycles are conflicting. Some of these studies have shown that the endometrial thickness is associated with serum E2 levels (8, 9), while others have failed to find a relationship between them (10, 11). We have found that the E2 levels increased in parallel with the endometrial thickness, but that the difference was not statistically significant. The reason for the discrepancy between spontaneous and stimulated cycles may be asynchrony in endometrial development and oocyte maturation (10). Unlike natural cycles, the ovarian steroid response and endometrial thickening do not occur in parallel in controlled hyperstimulation. This is consistent with the lack of a correlation found in our study between the number of retrieved oocytes and transferred embryos, and endometrial thickness.

Because progesterone is the hormone responsible for the secretory changes in the endometrium, we expected to find higher progesterone levels in the hyperechogenic group. Unexpectedly, it was insignificantly lower for the hyperechogenic pattern than the trilaminar or isoechogenic patterns. This is consistent with the results of other studies (4, 12). Although the exact mechanism is unknown, it is believed that other hormones, such as androgens and exogenous gonadotropins, cause premature echogenicity of the endometrium by direct effects on the endometrium (4).

In our study, the sonographic appearance of endometrium was evaluated by four specialists using the same ultrasound device. Although this is expected to affect the results, Spandorfer et al. have reported that there is excellent correlation between intraobserver and interobserver measurement of the endometrium (13).

## Conclusion

We showed that steroid levels on the day of hCG administration did not affect the ultrasound appearance of the endometrium. The thickness of the endometrium was not related to serum E2 levels. Different unknown mechanisms produced hyperechogenicity, other than premature exposure to progesterone. Further studies should be done to explore these mechanisms.
